# In Silico Investigation of the Interlaminar and Mechanical Fracture of Arteries with Atheromatic Plaque during Angioplasty Treatment

**DOI:** 10.3390/biomedicines12092105

**Published:** 2024-09-14

**Authors:** Spyridon Psarras, Anargyros-Nektarios Skafidas, Vassilis Kostopoulos

**Affiliations:** Department of Mechanical Engineering and Aeronautics, University of Patras, 26504 Patras, Greece

**Keywords:** angioplasty, stent, finite element, artery failure, interlaminar failure

## Abstract

The reduction in the inner diameter of the artery due to the creation of atheromatic plaque on the artery lumen, known as artery stenosis, disrupts the blood flow, leading to medical complications, which can be fatal. The angioplasty procedure aims to reopen the artery and uses a stent to keep it open. In this study, an effort is made to determine the point of the stent, the plaque and the artery during the expansion phase of the angioplasty using the in silico Finite Element Analysis method. A literature-based design was chosen for the stent geometry, whereas simplified shapes of the balloon and the two artery layers were used. Additionally, two plaque designs were the benchmark for the eight distinct artery stenosis models within the Abaqus environment. In the context of stent angioplasty simulations, failure patterns were investigated. An inverse relationship was observed between artery stenosis and pressure at the artery failure point, while an increased danger of interlaminar failure was detected in models with larger artery stenosis. This study verifies the necessity for the inclusion of interlaminar failure in future angioplasty research.

## 1. Introduction

Ischemic heart diseases are reported as the most common cause of death according to WHO reports [1]. To combat the stenosis of the arteries causing these diseases, angioplasty operations are performed by physicians. A wide variety of operations exists, with the most common being angioplasty with a stent. During this operation, a small cylindrical mesh placed on a balloon-catheter system is deployed at the stenosis region. Subsequently, the reopening of the artery is achieved while adding structural strength to the artery with the aid of the stent.

In addition to the improvement in the operation, due to the dependence of such operations on the expertise of doctors, a deeper comprehension of the procedures’ specific mechanisms is required. The Finite Element Analysis (FEA) method used within in silico models has proven itself a great tool compared to classic in vivo experiments by providing rapid results through multiple low-cost simulations. The first such effort was conducted by Migliavacca et al. [2], who provided an early optimal stent design, whereas a similar study by Martin D. and Boyle F. [3] investigated the balloon’s influence on the angioplasty procedure for the first time.

A general study for the more realistic depiction of the angioplasty procedure was conducted by Schiavone et al. [4]. According to their study, the results, resembling a real stent deployment, were extracted using a partially constrained artery with a layered structure of anisotropic layers and a folded balloon. On the other hand, the importance of the inclusion of the preoperative processes of balloon folding was highlighted by Geith et al. [5], attributed to the presence of the initial residual stresses cultivated during these processes. Additionally, the effect of tapered arteries on the angioplasty operation was explored by Shen et al. [6], demonstrating the importance of the balloon shape for the success of the operation.

Furthermore, studies where the FEA method is used to compare different stents provide information not easily found through in vivo studies. For instance, the Wiesent et al. [7] study used the dogboning ratio during stent deployment as a point of comparison between stent designs. A study by Umer et al. [8] provided a valuable comparison between stents under free and confined deployment, while Noble et al. [9], in a study comparing patient-specific arteries, discovered that plaque composition has a smaller impact on the operation when compared to the artery vessel shape. On the other hand, a rare comparison between computer models and laboratory experiments was achieved through an Antonini et al. [10] study, where the great accuracy of the computer models was confirmed. In order to execute the aforementioned studies in vivo, much more time and resources would have been needed, resulting in a higher cost of the study.

The use of bare metal stents in angioplasty, although simple, display clinical complications. The stent restenosis phenomenon found in post-operation examinations signify the need for a solution, which came in the form of drug coatings. When applied on the stent, a 60–80% reduction in in-stent restenosis cases is achieved [11]. Drug coating research also makes use of the FEA method. For instance, the transport and kinetics of the drug used for the coating were explored by Colombo et al. [12], while the effects of different coatings on the drug absorption variable were examined by Biswas et al. [13]. Similarly, a strong correlation between the drug absorption and the drug delivery amounts was found by a Jain et al. [14] study while investigating the influence of various parameters on the drug delivery process of a drug-coated balloon (DCB).

Such studies are not limited to investigating the pharmacokinetics of the drug, but also investigate the influence of the coating’s characteristics. The ideal diffusion coefficient value and coating thickness of a drug-eluted stent (DES) were identified by Li et al. [15] using the FEA method, whereas a bare metal stent and two stents with different coating thicknesses were compared by Loukas et al. [16] in FE models, thus evaluating coating influence on the stress and strain of the artery. Furthermore, while studying the contact pressure patterns between the DCB and the artery, Stratakos et al. [17] observed a notable inconsistency in the interaction between the balloon and the vessel, attributed to variations in the balloon’s unfolding process.

Additionally, in a rare FEA study by Escuer et al. [18], the effect of the artery curvature and the plaque composition on the drug delivery of a DES was investigated. Moreover, an interesting comparison between DCBs and DESs with different drug compositions was performed by Escuer et al. [19] where the superiority of the DCB and the DES over short-term and long-term results, respectively, were confirmed.

While the drug coatings successfully prevented stent restenosis, they failed to address other clinical complications such as stent thrombosis. To address that issue, the use of bioabsorbable materials has been suggested. The FEA method was first utilized for research on the subject by Wu et al. [20] for the optimization of the geometry of a magnesium-based bioresorbable stent (MAS). Additionally, another optimization effort was attempted by Li et al. [21], aiming to improve the MAS’s radial strength while also reducing the strut’s thickness.

Furthermore, a comparison between a bare metal stent and a magnesium-based stent was conducted by Alihemmati et al. [22] in an ideal artery–plaque geometry, revealing the need for increased stresses for the former to be deployed, increasing vascular injury risk. On the other hand, an extensive study on zinc-based bioresorbable stents was performed by Zhang et al. [23], focusing on the parameters influencing the degradation of these stents. A lot of potential is found in these bioabsorbable stents, although no extensive research has been conducted since, due to their recent development, FDA approval for these stents is rare [11]. In addition, the poor mechanical properties of zinc-based stents and the rapid degradation of MASs should be addressed before their wide adoption by the medical community.

Despite the technological advancements in the stent sector, rare clinical complications caused by artery rupture occur during 0.2–3% of angioplasty operations [24]. These complications should not be treated lightly, especially when for 33% of the cases with artery perforation, it led to the death of the patient [24,25,26,27,28]. The causes of these complications have not been adequately researched, linking many factors to their appearance [28]. Additionally, vessel rupture may not occur during the course of the operation but after its conclusion, such as the cases described by Broadbent P.L. et al. [26] and Chae et al. [27], which adds a layer of complexity to the problem. Attempts are made to further investigate plaque progression and its effect on artery fracture, such as that of the recent study by Mantzaris et al. [29], but the available data addressing the sources of blood vessel rupture are still scarce.

As such, the estimation of the mechanical and interlaminar limits of the arterial vessel during the angioplasty operation is of paramount significance. The aim of this study is to investigate the influence of stenosis on the interlaminar and mechanical rupture of the artery during the deployment phase of the angioplasty procedure.

## 2. Materials and Methods

The first step towards this goal is the creation of a digital simulation of the angioplasty deployment phase. The in silico models that were created consist of the stent, the balloon, the inner and outer artery layers, and two different plaque types. All models were created using the Abaqus 6.23 software, except for the stent that was imported from “step file” due to its complex shape.

### 2.1. Model Parts

**Figure 1:**
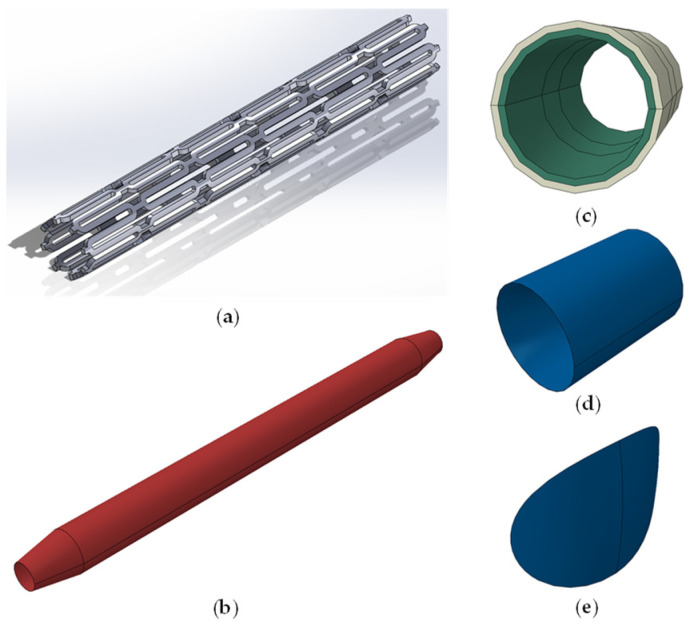
(**a**) SolidWorks stent model, (**b**) Abaqus balloon model, (**c**) Abaqus inner and outer artery layer models, (**d**) Abaqus quarter plaque model, (**e**) Abaqus cylindrical plaque model.

**Figure 2:**
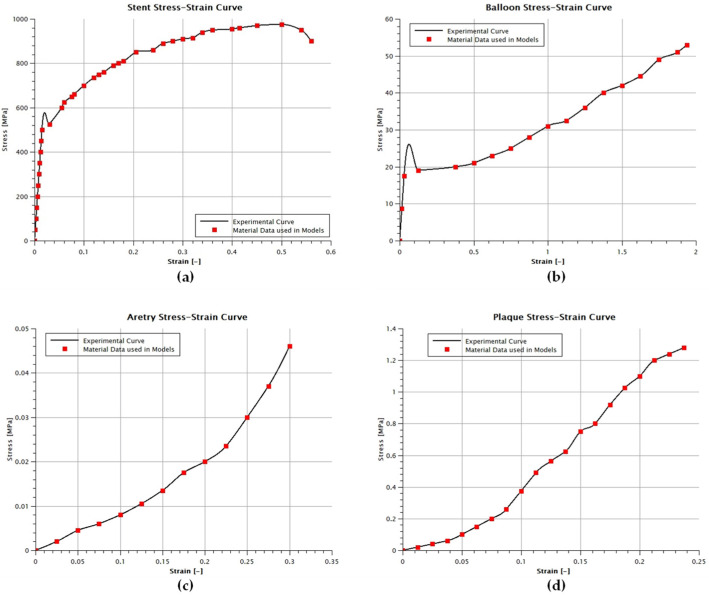
Material curves of (**a**) stent [35], (**b**) balloon, [37] (**c**) artery, [38] (**d**) plaque [41].

The geometry of the Cobalt–Chromium stent is based on the literature [30,31,32,33,34,35], and it consists of six rings, as seen in Figure 1a. The creation of the stent started with a single stent ring in a 2D depiction, followed by the “wrapping” of the 2D design around a cylinder with a diameter equal to the stent’s inner diameter. Afterwards, the wrapped design is extruded, resulting in a single stent ring, while the final stent is achieved by mirroring the single ring. The stent’s material characteristics were based on the literature-based curve [35] seen in Figure 2a. For the stent mesh, a C3D8R 8-node linear brick was used, while the material characteristics were extracted from the literature and are shown in Table 1.

The design of the balloon is based on previous studies [5,21,23,36] and consists of a cylinder and a cone base shape at the edges of the cylinder, as seen in Figure 1b. The total length of the balloon is 10 mm, and the diameter of the cylinder is equal to the stent’s inner one. Its material characteristics are drawn mainly from the stress–strain curve seen in Figure 2b. The mesh grid makes use of the S4R 4-node thin-shell elements, whereas the balloon’s mechanical characteristics are drawn from mechanical tests of the common balloon material polyamide 12 [37].

**Table 1:** Stent, artery, and plaque material properties.

Part	Young’s Modulus (MPa)	Poisson’s Ratio (-)	Yield Strength (MPa)	Ultimate Tensile Stress (MPa)	Density (kg/m^3^)
Stent [30,31,32,33,34]	33 × 10^3^	0.3	507.5	1404	8430
Artery [38,39,40]	9.56 × 10^−2^	0.49	0.88 × 10^−2^	5.98 × 10^−2^	1050
Plaque [40,41,42]	1.69	0.49	62.25 × 10^−3^	1.58	2700

The artery, consisting of two layers shown in Figure 1c, is modeled as two hollow cylinders of 15 mm in length. The radius of the inner wall surface is 1.25 mm, the outer wall radius is equal to 1.75 mm, and each layer has a 0.25 mm thickness. All geometry dimensions were drawn from the literature [6,16,22,43,44,45], while its material properties were mainly drawn from the stress–strain curve seen in Figure 2c. The C3D8R quad element was used for the arterial layer’s FE mesh, with their mechanical properties being based on the stress–stretch curve of the median layer, and Poisson’s ratio and the density were derived from other independent studies [38,39,40].

This study requires the creation of models which employ similar plaque geometries but result in different artery stenoses. To achieve that, based on the literature [6,22,43], two ideal different plaque geometry categories were created for the simulation: the cylindrical category and the quarter plaque category, shown in Figure 1d,e, respectively. Each category includes plaques of similar shape, but of different sizes, thus achieving the required different stenosis percentages.

The cylindrical plaques have a cylindrical shape, with their cross-section being a circle segment, and they are placed in the center of the artery, in accordance with similar studies which employ ideal artery and plaque shapes [6,22,43]. With their 3.5 mm length and varying maximum thicknesses, five cylindrical plaques were created, resulting in models with 7.84%, 15.36%, 36%, 48%, and 64% artery stenosis.

The quarter plaque category covers only a quarter of the arterial inner wall and has the shape of an ellipsoid in its inner area. Two quarter plaques of different sizes were created, from which three models were developed. The first two, with 7% and 17% stenosis, use a single smaller and a single larger quarter plaque, respectively. The last model, using a combination of two smaller quarter plaques and a single larger quarter plaque, results in a 34% stenosis.

The cylindrical plaque meshes are modeled using the C3D8R quad element, while the C3D10M tetrahedron element is adopted for the quarter plaque meshes due to their increased curved surfaces. Additionally, a calcified plaque material is chosen for the plaques, with its stress–stretch curve providing, as shown in Figure 2d, its mechanical properties. Once again, Poisson’s ratio and the density are derived from different studies [40,41,42].

### 2.2. Assembled Models

**Figure 3:**
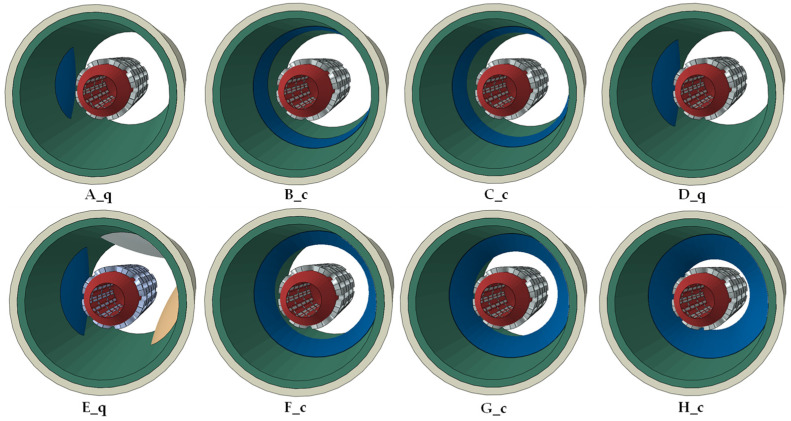
Aspects of all models. The primary lettering of the models shows the increasing stenosis percentage, while the secondary letter shows whether the model uses quarter or cylindrical plaques.

Using the previously mentioned parts, a total of eight models of increasing stenosis are created, shown in Figure 3. Aside from numbering the models using letters, a secondary letter is used to specify whether the model uses a cylindrical plaque or a quarter plaque. For example, model A_q is the first model with the smallest stenosis, and has a quarter plaque, while model B_c has the smallest stenosis among the cylindrical plaque models. In all models, the plaques are placed in the middle of the artery. This does not apply to model E_q, where the larger quarter plaque is placed in the middle of the artery, and the two smaller plaques are placed at a 157° and 255° angle relative to the quarter plaque and at a +0.65 mm and −0.30 mm distance from the middle of the artery.

In all of the models, the stents and the balloons are placed coaxially to the arterial layers and at the center of the vessel. The edges of the vessel layers and of the balloon are locked in place using a boundary condition, whereas the plaque outer surface is coupled with the central inner surface of the artery inner layer. The external loading is simulated by a displacement-driven boundary condition at the inner surface of the cylindrical balloon section. A smooth-step amplitude is selected for the external loading to achieve a more accurate model response, and a 3 mm balloon expansion is picked. It should be noted that the balloon expansion is much larger than the one employed in the actual operation. This choice was made to test the limits of the artery and the stent, while the balloon mechanical limits were not in the scope of this study.

Between the model parts’ surfaces, a frictionless interaction is imposed with the sole exception being the interaction between the outer surface of the inner vessel layer and the inner surface of the outer vessel layer. Among them, in order to replicate the interface between the artery layers, a hard-contact normal behavior is chosen, with the interface damage being simulated by the bilinear Traction Separation Law. Using this fracture criterion, a non-physical layer of cohesive elements is placed among the two surfaces, allowing the development of cohesive forces during the extension of the crack tip [46]. The traction elastic parameters and the nominal stresses that are used in the aforementioned models are shown in Table 2 [47,48].

In all of the aforementioned material properties and model parameters, a lack of time-dependable variables is observed. Due to the study being a simulation of catastrophic testing, in accordance with the similar literature [6,7,9,10,22], the time variable was deemed inconsequential to the models’ results. Subsequently, no time-dependent variables were included in the study.

### 2.3. Methodology

A number of failure types can be found in the models. Mechanical failure can be observed at the stent, at the plaque, or at the artery layers. Balloon failure cannot be taken into account due to the direct influence of the displacement-driven boundary condition/external loading leading to a non-realistic response during the course of the simulation. Additionally, the interlaminar arterial failure can be tracked using the damage initiation criterion of the Traction Separation Law for the cohesive interface between the artery layers, although in the model the interface has been defined as an interaction property.

Using the simulations, the stent radius can be tracked using a set of balloon nodes that come in contact with the inner strut of the stent, specifically created for that purpose. Additionally, a comparison between the stent and the artery radius is conducted in order to better understand by how much the inner lumen diameter has reached or surpassed its initial diameter. This is achieved by calculating the following “Comparison Value”, named CV for the purposes of this study:

CV = (R_stent_ − R_Artery-Inner_)/R_Artery-Inner_ 100 [%] (1)

The radius of the stent, being equal to the displacement-driven balloon displacement, displays the active radius of the artery when the stent has come in contact with the artery lumen. Subsequently, the CV can be viewed as an expression of the expansion of the artery as a percentage of the starting artery radius.

## 3. Results

### 3.1. Simulation Phases

**Figure 4:**
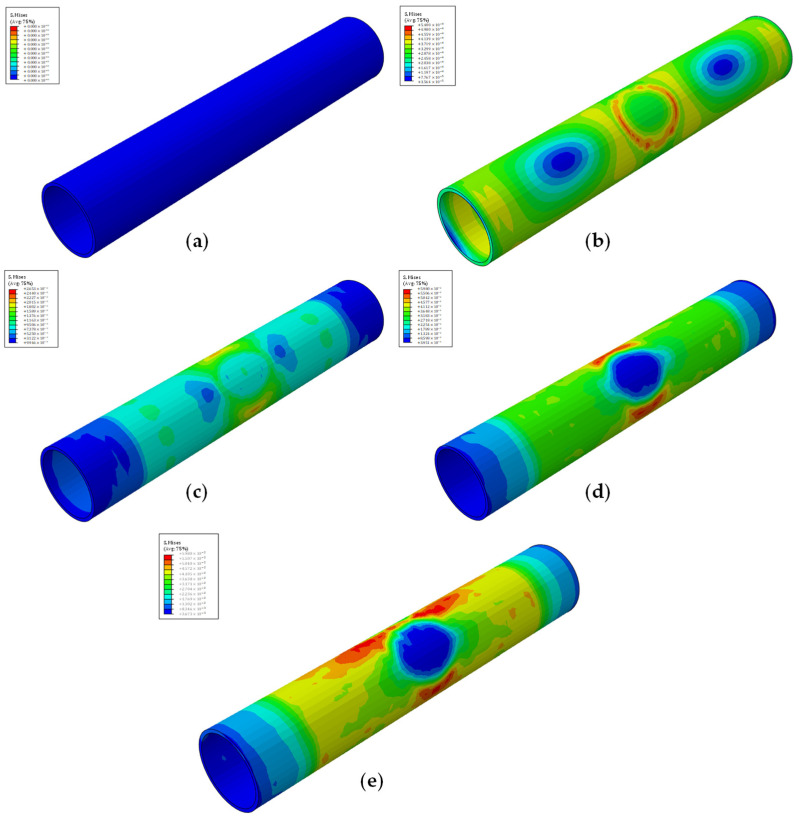
Simulation phases of an artery with quarter plaque. (**a**) Beginning of simulation; (**b**) stent and plaque first contact; (**c**) stent and artery direct contact; (**d**) damage initiation of artery; (**e**) damage propagation and end of simulation. Each phase has its own color map.

**Figure 5:**
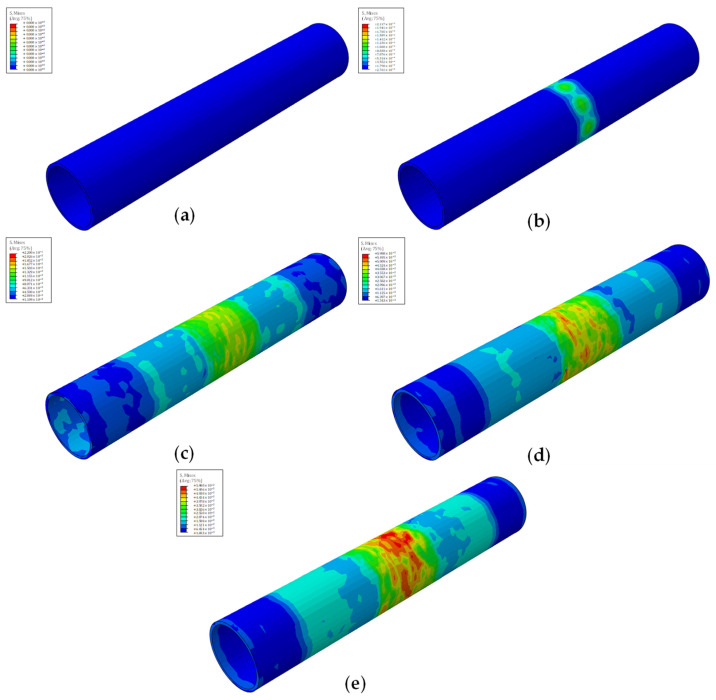
Simulation phases of an artery with cylindrical plaque. (**a**) Beginning of simulation; (**b**) stent and plaque first contact; (**c**) stent and artery direct contact; (**d**) damage initiation of artery; (**e**) damage propagation and end of simulation. Each phase has its own color map.

All models, despite capitalizing on different stenosis percentages and plaque shapes, exhibit similar behavior during the course of the simulations, displayed in Figure 4 for the quarter plaque models and Figure 5 for the cylindrical plaque models. After the start of each simulation, a first contact between the stent and the plaque is achieved, followed by direct contact between the stent and the artery. Afterward, damage initiation of the artery takes place, while damage propagation is tracked until massive vessel deformations are observed, at which point the simulation is terminated.

For models employing the quarter plaque, the maximum stresses are observed at the edges of the artery–plaque contact area, while damage initiation takes place specifically at the edge of the contact area of the central artery section or, for the case of model E_q, in the area between the plaque contact areas.

A slightly distinct behavior is observed in models which employ cylindrical plaque due to the plaque’s vastly different geometry. In contrast to the previous models, the maximum stresses during the course of the simulation are observed at the middle section of the vessel, clearly visible in Figure 5, which coincides with the middle of the artery–plaque contact area. The source of the behavior lies in the symmetry of the cylindrical plaque, due to which the artery stresses are evenly distributed around the central ring section of the artery. Over the course of the simulation, the stresses are evenly distributed around the perimeter of the arterial wall, with the maximum stresses being observed at the center and decreasing across the vessel’s length, with its edges displaying negligible stresses.

### 3.2. Angioplasty Failures in the Simulations

With the completion of the simulations, the failure types, as described in Section 2.3, are tracked and displayed in Table 3. Based on the simulations’ results, it is apparent that in all models, no plaque failure takes place, mostly due to the plaque’s greater ultimate tensile stress when compared to the artery’s. Specifically, the plaque’s tensile strength is more than 250% bigger than the vessel’s, inevitably resulting in the simulation’s termination due to artery failure being much earlier than the point of plaque failure. Thus, no threat of plaque failure is present over the course of the operation, and, subsequently, it is not included in the failure results tables.

**Figure 6:**
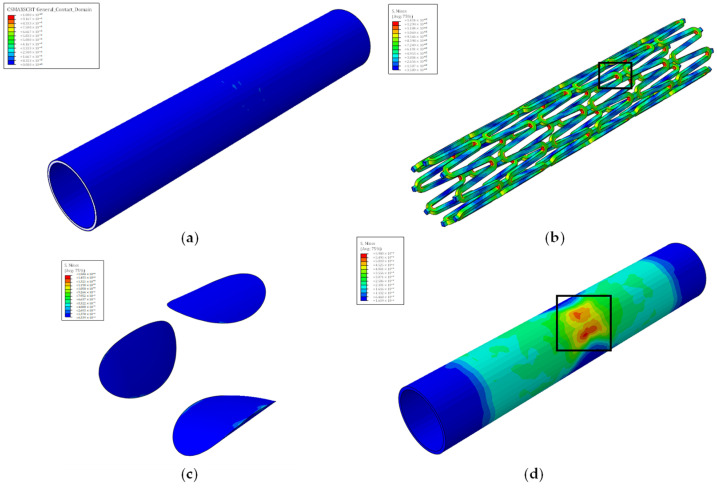
Detection of failures in a model with quarter plaques using stress plots. (**a**) Interlaminar failure; (**b**) stent mechanical failure; (**c**) plaque mechanical failure; (**d**) artery mechanical failure. For each part, the ultimate tensile strength is displayed by the red color. The failure area of each part can be seen in the black boxes.

Furthermore, stent failure occurs at a constant stent radius in all models. Specifically, stent failure happens when the radius is equal to 2.53 mm. As seen in Figure 6, stent failure occurs at its crown region, where an increased stress concentration factor occurs from the curved geometry.

**Figure 7:**
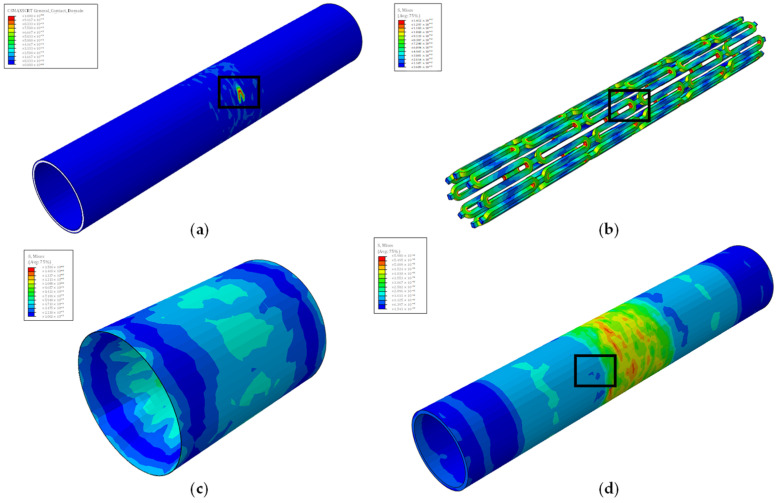
Detection of failures in a model with cylindrical plaque using stress plots. (**a**) Interlaminar failure; (**b**) stent mechanical failure; (**c**) plaque mechanical failure; (**d**) artery mechanical failure. For each part, the ultimate tensile strength is displayed by the red color. The failure area of each part can be seen in the black boxes.

All models display mechanical failure of the artery but at different stent radiuses. Interestingly, models using the quarter plaque (A_q, D_q, and Ε_q) display no interlaminar failure between the vessel wall layers, as seen in Figure 6, whereas in the cylindrical plaque models (B_c, C_c, F_c, G_c, H_c), interlaminar failure is observed, as shown in Figure 7.

Furthermore, by studying Table 3’s results of models with similar stenosis, in addition to the previous remark, a significant discrepancy is observed between the values of arterial failure. Specifically, between models A_q and B_c with a 0.6% difference in stenosis, C_c and D_q with a 1.4% stenosis difference, and E_q and F_c with a 2.0% stenosis difference, a 12.3%, 5.6%, and 10.9% difference in mechanical failure of the artery is observed. As a result, it is apparent that the plaque’s shape greatly influences the vessel’s behavior during the angioplasty procedure.

It should be noted that between models E_q and F_c, the mechanical failure of the artery is identical, as seen in Table 3. But, due to their different plaque shapes, in the model with the cylindrical plaque (F_c), interlaminar failure precedes mechanical failure. As such, the two models display artery failure at different stent radiuses and due to different causes, despite having a similar stenosis. This phenomenon highlights the effect of the plaque’s geometry on the artery’s behavior.

## 4. Discussion

Using the methodology described in Section 2.3, the comparison values (CVs) between the stent and the inner healthy artery radiuses are computed and are shown in Table 4.

From the CVs, it can be confidently inferred that stent failure is not a risk during the angioplasty procedure. This is obvious from the values of Table 4, where stent failure occurs when the CV is 240.5%, signifying that the inner artery radius at the point of stent failure is more than double the radius of the pre-operation healthy inner artery. During the procedure, achieving such a substantial artery expansion is unnecessary because the desired artery opening can be attained with stent deployment at much lower CVs. As such, the point of stent failure is never reached.

Concerning artery failures, an inverse relation is established between the pressure point of arterial failure (both mechanical and interlaminar) and the stenosis percentage. For models sharing the plaque type, an increase in stenosis was observed to lead to smaller CVs at the point of failure. Specifically, between the models with quarter plaque, concerning the comparisons of models A_q and D_q, as well as models D_q and E_q, the CV at the failure point decreases by 33.1% and 6.7%, respectively, as the stenosis increases by 132.2% and 102.5%, respectively. Additionally, for the cylindrical plaque models, between models B_c and C_c, models C_c and F_c, models F_c and G_c, and models G_c and H_c, for a 95.9%, 134.4%, 33%, and 33% increase in stenosis, respectively, the CV at the point of artery rupture shows a 21.6%, 21.9%, 2.7%, and 28.2% drop, respectively.

Finally, for the cylindrical plaque models, a very interesting phenomenon is observed. In model C_c, interlaminar failure of the artery occurs at a 2.6 mm stent radius, after its mechanical failure, which occurs at 2.0 mm. On the other hand, in model F_c, interlaminar failure occurs at 1.5 mm stent radius, before the vessel’s mechanical failure, which occurs at 1.8 mm. Additionally, in model B_c, a cylindrical model with a smaller stenosis than model C_c, the mechanical failure of the artery (at 2.7 mm) precedes the interlaminar failure (at 3.2 mm). The opposite occurs in cylindrical models with stenosis greater than model F_c’s stenosis, where interlaminar failure (at a 1.4 mm and 0.9 mm stent radius for models G_c and H_c, respectively) precedes mechanical failure (at a 1.5 mm and 1.0 mm stent radius for models G_c and H_c, respectively).

**Figure 8:**
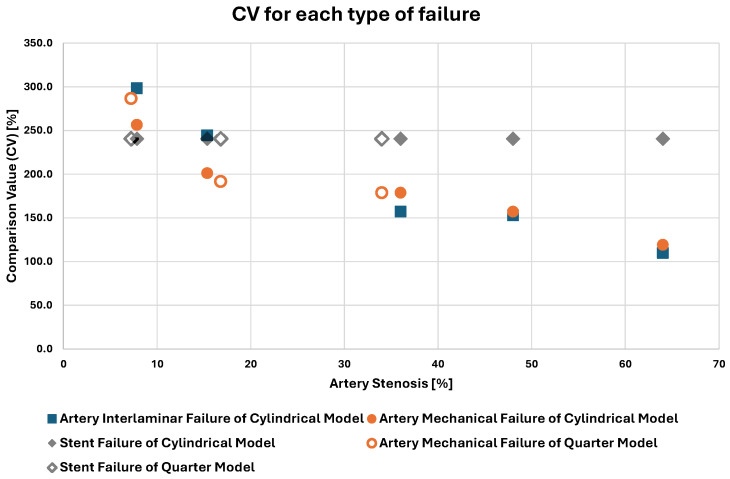
CVs for each type of failure for the different cylindrical model stenoses.

This observation, clearly seen in Figure 8, is of great importance. It signifies that after a certain “critical” stenosis percentage, interlaminar failure of the artery will occur before any mechanical failure occurs. According to the simulation, for the cylindrical plaque, this critical stenosis percentage is between 15.4% and 36% stenosis. While mechanical failure of the artery can be easily observed during the angioplasty operation, the same cannot be said about interlaminar failure. The subtle nature of the latter makes it very elusive to track down and difficult for physicians to anticipate. As seen in Figure 7, interlaminar failure is very small, and the area around seems to be very healthy. In contrast, the damage initiation area of mechanical failure is also small, but the area around it shows greater stresses, making it more apparent. As a result, interlaminar failure can be abruptly caused, leading to clinical complications such as the cases described by Vavuranakis et al. [24], Alfonso et al. [25], and Ekici et al. [28]. Even worse, it can be easily missed during the angioplasty operation, deeming the operation successful with no complications, while a post-procedure check shows a complication out of thin air, such as the cases described by Broadbent P.L. et al. [26] and Chae et al. [27]. This “critical” stenosis percentage differs between plaque shapes, as seen by the quarter plaques where no interlaminar failure occurs.

## 5. Conclusions

This study employed the FEA method to identify the critical point of arterial failure during the angioplasty operation for various degrees of arterial stenosis. Although idealized geometries were employed and the material properties of the artery and the plaque were obtained from various literature sources, the results drawn from the comparison of the simulations display a number of important findings.

Firstly, the study clearly showed that calcified plaque failure and stent failure are not of concern during angioplasty operations, as such failures either do not occur in the case of the plaques or take place at very large displacements in the case of the stents. Additionally, for a specific stenosis percentage, the divergence of artery failure due to different plaque geometries was confirmed. Moreover, the study revealed an inverse relation between the CV and artery stenosis percentage, where for a higher degree of stenosis, a lower CV was needed to achieve vessel failure, which was consistent with the intuitive expectation.

On top of the previous remarks, the existence of a “critical” stenosis percentage, after which interlaminar failure of the artery vessel becomes the prevalent failure, is very important. Such a discovery highlights the danger interlaminar failure can pose to the operation. Furthermore, it can explain post-procedure complications which had not occurred during or immediately after the operation due to the difficulty of detecting interlaminar failure before extensive damage propagation has taken place [24,25,26,27,28]. Further studies must be redirected to exploring the “critical” stenosis percentage of various plaque geometries. Specifically, due to the unique characteristic of each patient-specific artery, researchers should use such artery models to evaluate the danger of arterial interlaminar failure.

## Figures and Tables

**Table 2:** Parameters used to define the arterial layer interface [47,48].

Traction Elastic Parameters		E/E_nn_ [N/mm]	G_1_/E_ss_ [N/mm]	G_2_/E_tt_ [N/mm]
		4	4	4
**Damage Initiation Criteria**	**Normal-only Mode** **[MPa]**	**First Direction [MPa]**	**Second Direction [MPa]**	**Fracture Energy [kJ/m^2^]**
	0.213	0.324	0.324	1.210

**Table 3:** Stent radius for each type of failure per model.

MODEL *	Volume Stenosis [%]	Stent Radius at the Point of Failure Due to		
		Interlaminar Failure [mm]	Artery Mechanical Failure [mm]	Stent Failure [mm]
A_q	7.2	-	3.1	2.5
B_c	7.8	3.2	2.7	2.5
C_c	15.4	2.6	2.0	2.5
D_q	16.8	-	1.9	2.5
E_q	34	-	1.8	2.5
F_c	36	1.5	1.8	2.5
G_c	48	1.4	1.5	2.5
H_c	64	0.9	1.0	2.5

* Models with “q” sublabel refer to models using quarter plaque, while models with “c” sublabel refer to models using cylindrical plaque.

**Table 4:** Comparison value (CV) for each type of failure per model.

MODEL *	Volume Stenosis [%]	Comparison Value (CV) for Failure Due to		
		Interlaminar Failure [%]	Artery Mechanical Failure [%]	Stent Failure [%]
A_q	7.2	-	286.5	240.5
B_c	7.8	298.4	256.5	240.5
C_c	15.4	244.6	201.2	240.5
D_q	16.8	-	191.7	240.5
E_q	34	-	178.8	240.5
F_c	36	157.1	178.8	240.5
G_c	48	152.9	157.1	240.5
H_c	64	109.8	119.1	240.5

* Models with “q” sublabel refer to models using quarter plaque, while models with “c” sublabel refer to models using cylindrical plaque.

## Data Availability

The data sets generated during and/or analyzed during the current study are not publicly available but are available from the corresponding authors on reasonable request.
